# Thymopoiesis, Alterations in Dendritic Cells and Tregs, and Reduced T Cell Activation in Successful Extracorporeal Photopheresis Treatment of GVHD

**DOI:** 10.1007/s10875-021-00991-y

**Published:** 2021-03-02

**Authors:** Aisling M. Flinn, Anna Ehrlich, Catherine Roberts, Xiao Nong Wang, Janet Chou, Andrew R. Gennery

**Affiliations:** 1 Translational and Clinical Research Institute, Newcastle University, Newcastle upon Tyne, UK; 2 Great North Children’s Hospital, Newcastle upon Tyne, UK; 3 Division of Immunology, Boston Children’s Hospital, Harvard Medical School, Boston, MA USA

**Keywords:** Extracorporeal photopheresis, acute graft-versus-host disease, T cell, thymus, dendritic cell, regulatory T cell

## Abstract

Acute graft-versus-host disease (aGVHD) is a significant complication of allogeneic hematopoietic stem cell transplant (HSCT) and negatively affects T cell reconstitution. Extracorporeal photopheresis (ECP) reduces aGVHD, but the mechanisms remain incompletely understood. Our objective was to examine the impact of ECP on thymopoiesis in pediatric aGVHD and the mechanisms at a cellular and transcriptional level. Sixteen pediatric HSCT patients were recruited: 6 with ECP-treated aGVHD, 5 without aGVHD, and 5 with aGVHD treated with corticosteroids only. Thymopoiesis was evaluated by measuring naive T cells, TRECs, IL-7, and T cell receptor repertoire diversity. Regulatory T cell (Treg) enumeration and function and dendritic cell (DC) enumeration and phenotype were analyzed using flow cytometry. T cell transcriptome analysis was performed on ECP patients after treatment and responders pre- and post-treatment. Four ECP responders demonstrated thymic-dependent T cell recovery, and superior median naïve T cell numbers at 8 and 12 months post-HSCT compared to the aGVHD corticosteroid group. Increased Tregs and Treg suppressive function, reduced cDC/pDC and DC co-stimulatory marker expression in ECP responders suggest upregulated peripheral tolerance; these findings were not observed in partial responders. Responder post-ECP CD3+ T cell transcriptional profile demonstrated 3333 downregulated and 364 upregulated genes, with significant downregulation of ERRα and GαS pathways, and reduced expression of pro-inflammatory and adhesion proteins.

Thymic function improves with successful ECP treatment. ECP reduces T cell activation and impacts peripheral tolerance via DCs and Tregs. Differences in thymic recovery, DC, and Treg cellular patterns and the T cell transcriptome were observed between ECP responders and partial responders and require further validation and investigation in additional patients.

**Supplementary Information:**

The online version contains supplementary material available at 10.1007/s10875-021-00991-y.

## Introduction

Acute graft-versus-host disease (aGVHD) is a major complication of allogeneic hematopoietic stem cell transplantation (HSCT). T cell reconstitution is essential for a successful HSCT outcome but is negatively affected by aGVHD and its first-line treatment, corticosteroids [1–6] (Fig. S1). Corticosteroid-refractory disease often requires additional immunosuppressive agents creating a precarious balance between controlling aGVHD and the risk of infection and relapse.

Extracorporeal photopheresis (ECP) is an alternative therapy for aGVHD with a well-established clinical efficacy and safety profile [7, 8]. ECP involves collection of peripheral blood mononuclear cells (PBMC) by apheresis, exposure to 8-methoxypsoralen, and ultraviolet A radiation, followed by re-infusion of photoactivated cells into the patient. Uniquely, there are no systemic immunosuppressive effects and ECP facilitates weaning of immunosuppressive medications; therefore, ECP does not increase infection or disease relapse risk [9, 10]. Exactly how ECP exerts its therapeutic effects is not completely understood, but several mechanisms contributing to antigen-specific immunomodulation have been proposed including apoptosis of exposed lymphocytes, differentiation of monocytes into dendritic cells (DC) stimulated by physical interactions within the ECP chamber, promotion of a tolerogenic DC phenotype and increased regulatory T cells (Treg) although many studies incorporate in vitro ECP models or animal models [11–15]. Retrospective studies also suggest that ECP can promote thymic-dependent T cell recovery post-HSCT [3, 16]. We previously observed that ECP-treated patients demonstrated limited thymic recovery, although ECP was commenced late in the course of aGVHD and earlier initiation may further improve thymic output [3].

We aimed to prospectively examine the impact of ECP on thymic-dependent T cell recovery in a pediatric cohort by quantitative (naive T cell and TREC enumeration) and qualitative (T cell receptor (TCR) repertoire diversity) analyses. IL-7, a cytokine produced by thymic epithelial cells in response to lymphopenia [17], was measured as an indicator of functioning thymic stroma. We hypothesized that patients who respond to ECP have successful return of quantitative and qualitative thymic function. We aimed to explore ECP mechanisms in promoting immunotolerance by analysis of Tregs and DCs throughout treatment and T cell transcriptome analysis before and after treatment. We hypothesized that we would observe alterations in Tregs and DCs (consistent with increased peripheral tolerance) and the T cell transcriptome in patients with treatment progression and we would identify differences between those who respond successfully to ECP and those who fail to respond.

## Methods

### Study Participants

This was an observational exploratory study involving allogeneic HSCT pediatric patients who received ECP treatment for aGVHD, following initial corticosteroid treatment. In addition, two control groups were recruited; pediatric allogeneic HSCT patients who did not develop aGVHD (“no aGVHD” group) and pediatric allogeneic HSCT patients with aGVHD treated with systemic corticosteroids but who did not receive ECP (“aGVHD corticosteroid” group). Patients were excluded from recruitment to the “no aGVHD” group if they received corticosteroids for another clinical indication. Decision to treat with ECP and weaning of immunosuppression was a physician-led clinical judgment. Patients were recruited from June 2016–January 2018. All patients attended the Great North Children’s Hospital, Newcastle upon Tyne, UK. Clinical data recorded included demographics, underlying diagnosis, details regarding the HSCT and conditioning regimen, immunosuppressive agents used, type of GVHD, stage, and response to ECP. Grade of aGVHD was determined using the modified Glucksberg criteria. The NIH Consensus scoring system was used for ECP patient 5 with lung involvement [18]. Ethical approval was granted by the South Eastern Scotland Research Ethics Committee (16/SS/0019/AM03). Written informed consent was obtained from all patients or their legal guardians.

ECP was administered on 2 consecutive days weekly for the first 9 weeks, then every 2 weeks for 7 weeks, every 3 weeks for 4 weeks and monthly thereafter until treatment cessation, although some patients deviated from this protocol based on judgment of the attending physician. One ECP cycle was defined as two ECP procedures over two consecutive days. ECP was delivered using the CELLEX® System (Mallinckrodt Pharmaceuticals, NJ, USA). Patients were designated “ECP responders” if they completed ECP treatment with GVHD resolution and weaning of immunosuppression. Patients were designated “ECP partial responders” if they remained on ECP with partial clinical improvement.

For the ECP group, blood was taken on day 1 of each cycle, and at 4, 8, and 12 months post-HSCT. For the control groups, blood samples were taken at 4, 8, and 12 months post-HSCT. There were two missing samples at 8 months and two at 12 months follow-up in the “no aGVHD” group. In the “aGVHD corticosteroid” group, there was one missing sample at 8 months follow-up. There were no missing samples in the ECP group. Due to more limited sample volumes from the control groups, analyses were confined to naïve T cells, DCs, and Tregs.

### Flow Cytometry

For enumeration of cell absolute counts and frequencies, whole blood was stained with fluorochrome-conjugated antibodies in Trucount® tubes (BD Biosciences) and incubated for 20 min before adding 900 μl of red blood cell lysis buffer. For enumeration of naive T cells (CD3^+^CD4^+^CD45RA^+^CD31^+^), Tregs (CD3^+^CD4^+^CD25^hi^CD127^lo^), and activated T cells (CD3^+^CD4^+^HLA-DR^+^), the following antibodies were used (BD Biosciences): CD45-APC (HI30), CD3-Pe-Cy7 (SK7), CD4-PE (RPA-T4), CD45RA-BUV737 (HI100), CD31-APC Cy7 (WM59), HLA-DR-BUV395 (G46-6), CD127-BV421 (HIL-7R-M2), and CD25-FITC (M-A251) (Fig. S2). For DC enumeration (conventional DCs, cDCs; CD45^+^CD3^−^CD34^−^CD14^−^DR^+^CD4^+^CD16^−^CD11c^+^ and CD11c^−^CD123^+^ plasmacytoid DCs, pDCs), the following antibodies were used (BD Biosciences unless otherwise stated): CD3-FITC (UCHT1), CD4-PE (RPA-T4), CD16-PE-Dazzle (3G8, Biolegend), CD123-PerCP-Cy5.5 (7G3), CD45-AF700 (H130, Biolegend), CD34-APC-Cy7 (581, Biolegend), CD11c-BV421 (B-ly6), HLA-DR-V500 (G46-6), CD14-BV650 (M5E2) (Fig. S3). Data were collected using the Fortessa X-20 flow cytometer (BD Biosciences).

For DC phenotype analysis, PBMCs were prepared by density-gradient centrifugation using Lymphoprep® solution (STEMCELL Technologies) and incubated with the following antibodies (Biolegend unless otherwise stated): CD3-AF700 (SK7), CD19-AF-700 (HIB19), CD20-AF700 (2H7), CD14-BV650 (M5E2), CD11c-BV711 (3.9), HLA-DR-BUV395 (G46-6, BD Biosciences), CD16-PECF594 (3G8), CD123-BV785 (6H6), CD80-PE (L307.4, BD Biosciences), CD83-BV421 (HB15e, BD Biosciences), CD86-FITC (2331, BD Biosciences), and DAPI viability dye (Fig. S4). Data were collected using the BD FACSymphony flow cytometer (BD Biosciences). Median fluorescence intensity (MFI) was measured for CD80, CD83, and CD86 expression and the median value of the ECP responders (*n* = 4) and control groups (*n* = 3 in each) was calculated. Flow cytometry data were analyzed using the FlowJo software (BD Biosciences).

### Treg Suppression Assay

Patient PBMCs were stained with CD4-PE (RPA-T4), CD14-BV650 (M5E2), CD127-BV421 (HIL-7R-M21), CD25-FITC (M-A251), and DAPI, and CD25^hi^CD127^lo^ Tregs were sorted using the FACS-Fusion Sorter (BD Biosciences). Monocytes were isolated from healthy control PBMCs using a MACS separation system (Miltenyi Biotec) according to the manufacturer’s instructions. DCs were then generated by incubation of the isolated monocytes in RF-10 media with IL-4 and GM-CSF for 6 days followed by a further 24-h culture with 0.1 μg/ml LPS. Treg suppression capacity was measured in a co-culture system in which a CellTrace Violet™ (CTV) (ThermoFisher)–labeled effector T cells (Teffs) were cultured with allogeneic DCs (10:1 Teff to DC ratio, both from healthy controls) in the presence of ECP patient Tregs (4:1 Teff to Treg ratio). After 5 days, the frequency of proliferating Teff cells (CTV low) was detected using flow cytometry (BD Biosciences) (Fig. S5) at 3 time points of ECP treatment (early, mid, and late/end). Co-culture without Tregs served as control.

### TREC Quantification

T cell receptor excision circle (TREC) quantification was performed using a real-time PCR instrument (7900HT Fast Real-Time PCR System, Applied Biosystems) as previously described [19]. A standard curve was generated by cloning of plasmid TREC/TRAC DNA kindly supplied by Sottini et al [19] DNA was purified from 5 × 10^6^ PBMCs using the QIAamp® DNA Blood Mini Kit (Qiagen). The following primers and probes were used (Integrated DNA Technologies): TREC forward primer 5′-CAC ATC CCT TTC AAC CAT GCT-3′, TREC reverse primer 5′-TGC AGG TGC CTA TGC ATC A-3′, TREC probe 5′-FAM-ACA CCT CTG GTT TTT GTA AAG GTG CCC ACT-TAMRA-3′, TRAC forward primer 5′ TGG CCT AAC CCT GAT CCT CTT-3′, TRAC reverse primer 5′-GGA TTT AGA GCT TCT CAG CTG GTA CAC-3′ and TRAC probe 5′-FAM-TCC CAC AGA TAT CCA GAA CCC TGA CCC-TAMRA-3′. Data were analyzed using Sequence Detection Systems software version 2.4 (Applied Biosystems) and results were recorded as the number of TRECs per 1 ml of blood.

### T Cell Receptor Repertoire Analysis

T cell receptor (TCR) diversity was evaluated using complementarity-determining region 3 (CDR3) spectratyping. RNA was extracted from PBMCs using the RNeasy® Mini Kit (Qiagen), quantified using a NanoDrop® ND-1000 spectrophotometer (LabTech), and converted to cDNA using the High-Capacity cDNA Reverse Transcription Kit (Applied Biosystems). The TCRβ variable region was amplified using twenty-three Vβ primers (Fig. S6) followed by a run-off reaction with FAM-labeled Cβ primer and CDR3 fragment length analysis by capillary electrophoresis using the 3130 Genetic Analyzer (ThermoFisher). Data were analyzed using Peak Scanner (Applied Biosystems). Using healthy control samples, a scoring system was developed to define electropherograms as normal, abnormal, or highly abnormal (Fig. S7). Gaussian distributions were a subjective description of each Vβ family to demonstrate the visual change in the TCR repertoire, rather than a statistical description.

### Serum IL-7 Quantification

Serum IL-7 was quantified using the Quantikine® enzyme-linked immunosorbent assay (R&D Systems) as per the manufacturer’s instructions. Optimal density was measured using a Multiskan Ascent® plate reader (ThermoFisher) and data were analyzed by Ascent® software (ThermoFisher). Elevated IL-7 levels were defined as >9.8 pg/ml, as determined by the manufacturers.

### Whole Transcriptome Sequencing

Transcriptional analysis was performed on three ECP responders (patients 1, 2, and 4) and two partial responders (patients 5 and 6) pre- and post-ECP. Patients’ CD3^+^ T cells were purified by magnetic positive selection (Miltenyi Biotec), followed by RNA isolation with the RNeasy Micro Kit (Qiagen). cDNA was synthesized from 10 ng of total RNA using SuperScript™ VILO™ cDNA Synthesis Kit (ThermoFisher). The Ion AmpliSeq Transcriptome Human Gene Expression Kit was utilized to generate barcoded libraries per the manufacturer’s protocol and sequenced using an Ion S5TM system. Differential gene expression analysis was performed using the ampliSeqRNA plugin (ThermoFisher). Pathway analysis was done using Ingenuity Pathway Analysis (Qiagen).

### Data Analysis and Statistics

Longitudinal changes in naive T cells, TRECs, IL-7, TCR repertoire, DCs, and Tregs were measured over the course of ECP treatment. Naive T cell number, cDC/pDC ratio, and Treg number and frequency were compared with the control groups at 4, 8, and 12 months post-HSCT. Numerical data are reported as medians with ranges and mean values with error bars. Differences in median values were analyzed using the Kruskal-Wallis test, with a *p* value <0.05 being significant. Data were analyzed using GraphPad Prism.

## Results

### Patient Clinical Characteristics

Sixteen pediatric patients were recruited. There were 6 patients in the ECP group with steroid-refractory aGVHD and 5 patients in each of the “no aGVHD” and “aGVHD corticosteroid” control groups. A summary of the clinical characteristics and details of aGVHD diagnoses and management are provided in Tables 1 and 2 respectively.

**Table 1:** Summary of patient characteristics. Conditioning was described as myeloablative (MA) if cyclophosphamide was used in combination with busulfan or with total body and cranial irradiation, reduced toxicity MA if cyclophosphamide was used in combination with fludarabine, and reduced intensity conditioning (RIC) if fludarabine and treosulfan were used, with or without additional thiotepa

	ECP group, *N* = 6	No aGVHD group, *N* = 5	aGVHD corticosteroid group, *N* = 5
Age at HSCT (years)			
Median	7.7	4.3	6.6
Range	4.1–13	0.6–17.8	1.5–15
Gender			
Male	4 (66.7%)	2 (40%)	2 (40%)
Female	2 (33.3%)	3 (60%)	3 (60%)
Underlying diagnosis			
Immune deficiency	2 (33.3%), STAT3 GOF, CTLA4 deficiency	3 (60%), Nijmegen-breakage syndrome, CGD, SCID	3 (60%), hyper IgD syndrome, STAT3 GOF, CGD
Malignancy/hematological	4 (66.7%), relapsed AML × 2, relapsed ALL, high-risk AML	2 (40%), severe congenital neutropenia, severe aplastic anemia	2 (40%), high-risk ALL, AML
HSCT source			
BM	4 (66.7%)	4 (80%)	2 (40%)
PBSC	2 (33.3%)	1 (20%)	3 (60%)
HSCT donor			
Sibling/MFD	3 (50%)	3 (60%)	0
MUD	3 (50%)	1 (20%)	4 (80%)
Haploidentical	0	1 (20%)	1 (20%)
HLA Matching			
10/10	6 (100%)	4 (80%)	2 (40%)
<10/10	0	1 (20%)	3 (60%)
Conditioning			
MA	4 (66.7%)	0	2 (40%)
Reduced toxicity MA	0	1 (20%)	0
RIC	2 (33.3%)	4 (80%)	3 (60%)
TBI	1 (16.7%)	0	1 (20%)
Serotherapy	4 (66.7%)	4 (80%)	5 (100%)
GVHD prophylaxis			
CSA/MMF	2 (33.3%)	3 (60%)	2 (40%)
CSA alone	4 (66.7%)	1 (20%)	2 (40%)
None	0	1 (20%)	1 (20%)

*BM* bone marrow, *PBSC* peripheral blood stem cells, *MFD* matched family donor, *MUD* matched unrelated donor, *CSA* cyclosporin, *MMF* mycophenolate mofetil, *TBI* total body irradiation, *STAT3 GOF* signal transducer and activator of transcription 3 gain-of-function, *CGD* chronic granulomatous disease, *CTLA4* cytotoxic T lymphocyte antigen 4, *AML* acute myeloid leukemia, *ALL* acute lymphoblastic leukemia

**Table 2:** Details of patients with aGVHD treated with ECP. Overall grade of aGVHD was determined using the modified Glucksberg criteria. Therapies in italics denote continued aGVHD prophylaxis

ECP group						
Patient	GVHD organ involvement (max stage)	Max aGVHD grade	Treatment (excluding ECP)	Reason for ECP	Time from HSCT -ECP (days)	ECP outcome, number of ECP cycles
P1	GIT (3), skin (2)	3	CS, *CSA*, IFX	CS refractory	55	Complete response, 24
P2	GIT (3), skin (2)	3	CS, *CSA*, IFX	CS refractory	39	Complete response, 21
P3	GIT (3)	3	CS, CSA*, MMF, IFX	CS refractory	124	Complete response, 20
P4	Skin (3)	2	CS, CSA*, MMF, IFX	CS refractory, CMV viraemia	40	Complete response, 16
P5	Skin (3), liver (2), Lung (NIH score 3)	3	CS, *CSA, MMF*, IFX, BUD	CS refractory	173	Normalization of bilirubin, stable respiratory status (PFTs: FEV1 34%, FEV1/FVC 0.63), persistent exertional dyspnea, 44 (ongoing)
P6	Lung	-	CS, *CSA*, BUD	CS dependency	142	Improvement in PFTs; FEV1 35%, FEV1/FVC 0.91 pre-ECP to FEV1 69%, FEV1/FVC 0.98, persistent exertional dyspnea, 21 (ongoing)
aGVHD corticosteroid group						
Patient	aGVHD organ involvement (max stage)	Max grade	Treatment			Outcome
P1	Skin (3)	2	Topical + systemic CS, *CSA*			Complete response
P2	Skin (3)	2	Topical + systemic CS			Complete response
P3	Skin (3)	2	Topical + systemic CS, *CSA, MMFs*			Complete response
P4	Skin (3)	2	Topical + systemic CS, *CSA*			Complete response
P5	Skin (3)	2	Topical + systemic CS, *CSA, MMF*			Complete response

*BUD* budesonide (inhaled), *CMV* cytomegalovirus, *CS* corticosteroids, *CSA* cyclosporin, CSA* CSA later changed to tacrolimus, *FEV1* forced expiratory volume in one second, *FVC* forced vital capacity, *IFX* infliximab, *MMF* mycophenolate mofetil, *PFT* pulmonary function tests

This was the first HSCT for all patients and none received donor lymphocyte infusions. Median age at HSCT was lowest in the no aGVHD group (4.3 years) and highest in the ECP group (7.7 years). The majority of patients in each group received serotherapy and one patient from the ECP group and one from the aGVHD corticosteroid group received total body irradiation (14 Gy). Patients in the aGVHD corticosteroid group had grade 2 skin aGVHD and received treatment with topical and systemic corticosteroids, along with continuation of aGVHD prophylaxis.

### ECP Responders

ECP patients 1–4 had gut or skin aGVHD with a maximum grade of 2–3. All were refractory to corticosteroids; patient 4 also had cytomegaloviremia. Median time from HSCT to ECP commencement was 48 days (range 39–124 days). All patients had complete resolution of aGVHD with successful weaning of immunosuppression.

### ECP Partial Responders

Patients 5 and 6 demonstrated partial clinical improvement. Patient 5 initially developed corticosteroid-responsive skin aGVHD, followed by pneumonitis, which partially improved with corticosteroids and infliximab, alongside antimicrobial, and antifungal therapies. Thoracic computerized tomography (CT) demonstrated bronchiolitis obliterans with an obstructive pulmonary function test (PFT) pattern (FEV1 33%, FEV1/FVC 0.63). Lung biopsy was not performed due to poor clinical condition and ECP was started leading to initial clinical improvement. Upon weaning of ECP to four weekly and corticosteroids to 0.1 mg/kg at cycle 30, patient 5 developed liver aGVHD (biopsy confirmed). Weekly ECP and high dose corticosteroids were re-initiated and sirolimus was started, leading to normalization of bilirubin, and respiratory status remained stable (FEV1 34%, FEV1/FVC 0.63, persistent exertional dyspnea). Patient 5 was considered to have overlap syndrome, with features of aGVHD present but lung involvement distinctive of chronic GVHD (cGVHD) (NIH score 3), although no other cGVHD features were present. Patient 6 developed cough and dyspnea with a restrictive PFT pattern (FEV1 35%, FEV1/FVC 0.91) day +39 post-HSCT. Chest CT demonstrated diffuse pulmonary nodularity and lung biopsy identified active mild CD3^+^ lymphocytic bronchiolitis, consistent with partially treated aGVHD. No infectious causes were identified. Due to steroid dependency, ECP was commenced leading to PFT improvement (FEV1 69%, FEV1/FVC 0.98). Patient 6 had no other aGVHD organ involvement and no features of cGVHD.

### Thymic-Dependent T Cell Recovery

**Fig. 1:**
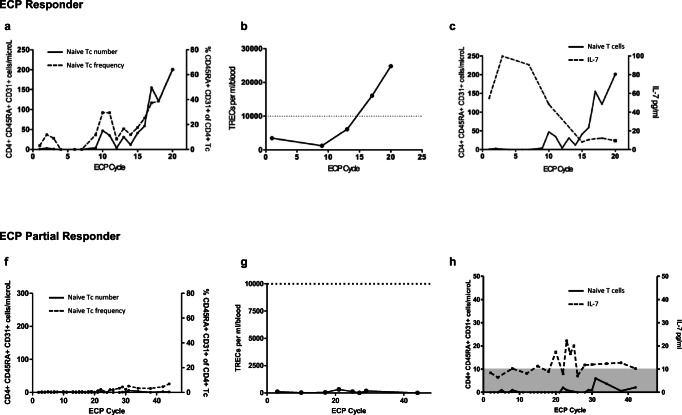

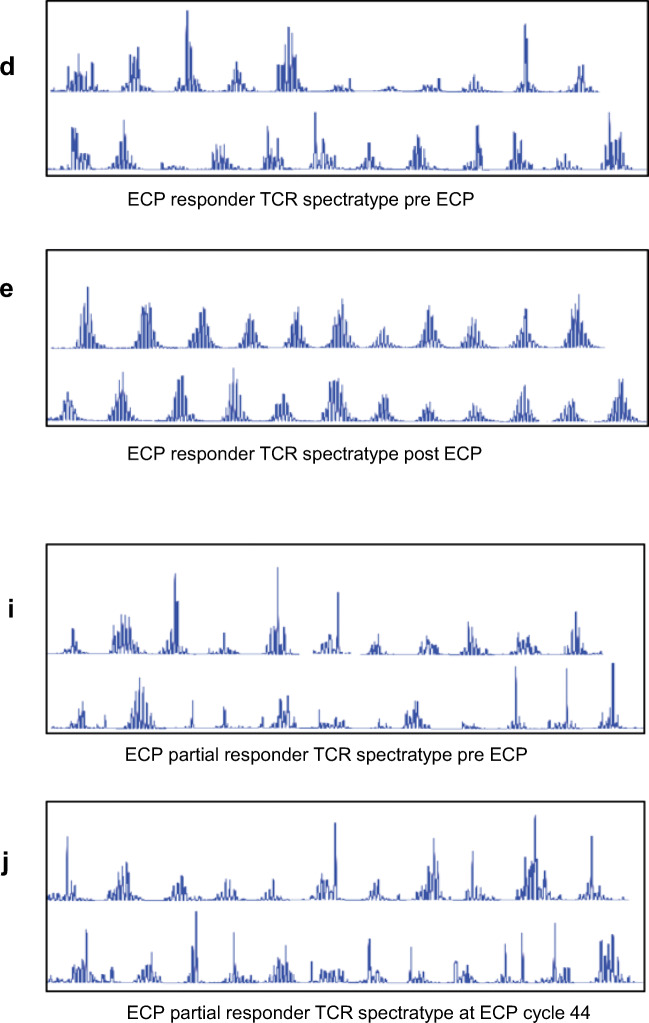

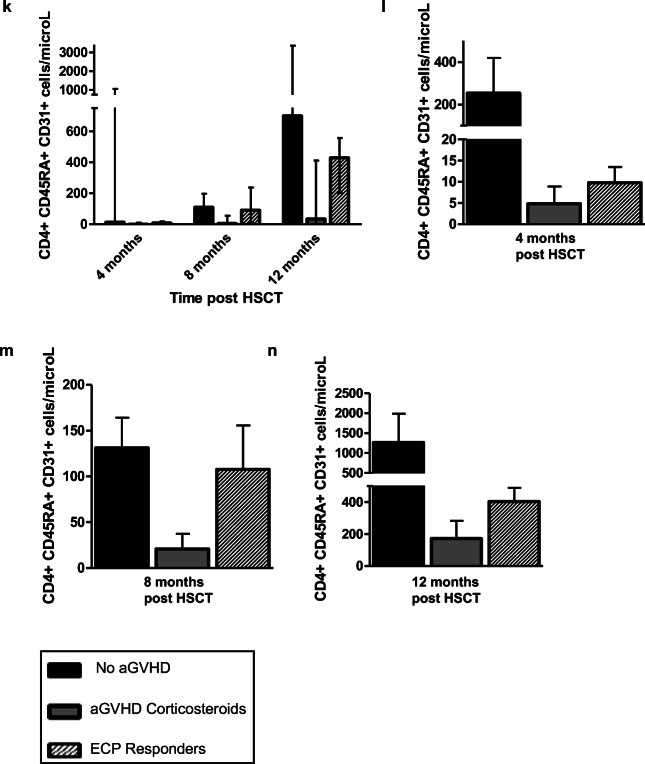
In the ECP responders (example shown is patient 3), **a** increased frequency (dotted line) and number (continuous line) of CD4^+^CD45RA^+^CD31^+^-naive T cells and **b** TRECs (dashed line represents the upper end of the normal range for age) were observed. **c** An inverse relationship between serum IL-7 (dotted black line) and naive T cells (continuous line) was seen. **d**, **e** Evaluation of the TCR repertoire indicates qualitative T cell improvement post-ECP as demonstrated by all TCR Vβ families present with a normal Gaussian distribution, compared to pre-ECP when several non-Gaussian families were seen with multiple monoclonal peaks. Partial responder 5 demonstrated **f** ongoing negligible number (continuous line) and frequency (dotted line) of naive T cells and **g** TRECs. **h** Increased IL-7 (dotted line) was not sustained and an inverse relationship with naive T cells (continuous line) was not evident. **i**, **j** Qualitative T cell improvement was not observed with a persistently abnormal TCR repertoire later in the course of ECP. **k** Comparison of median numbers (with range) of naive T cells at 4, 8, and 12 months post-HSCT demonstrate fastest thymic-dependent T cell recovery in patients with no aGVHD or additional immune suppression. Median naive T cell number was superior in the ECP responder group compared to the aGVHD corticosteroid group at 8 and 12 months post-HSCT. Differences between the groups were not statistically significant (*p* value = 0.25). **l**–**n** Mean numbers of naive T cells (error bars indicating SEM) at 4, 8, and 12-months post-HSCT respectively demonstrate a similar pattern with highest numbers in patients with no aGVHD

All patients exhibited low naive T cells and TRECs, and abnormal TCR repertoires at commencement of ECP regardless of timing post-HSCT, indicating poor thymic output. Increased naive T cells and TRECs, and an inverse relationship with IL-7, was observed with treatment progression in ECP responders. (Fig. 1a–c, Fig. S8). This coincided with resolution of clinical aGVHD, weaning of immunosuppression, and decreased activated T cell frequency (Fig. S9). Improvement in TCR diversity illustrated qualitative T cell improvement (Fig. 1d–e, Table S1). These results indicate thymic-dependent T cell recovery during ECP treatment. In contrast, partial responders 5 and 6 demonstrated no thymic-dependent T cell recovery 2 years post-HSCT and 44 ECP cycles, and 1 year post-HSCT and 21 ECP cycles respectively. An inverse relationship with IL-7 was not observed (Fig. 1f–j, Fig. S8).

To examine how thymic-dependent T cell recovery in the ECP responders compared with the normal trajectory post-HSCT, results were compared with the no aGVHD and aGVHD corticosteroid groups at 4, 8, and 12 months following HSCT (Fig. 1k–n). Thymic-dependent T cell recovery was superior in the no aGVHD group at each time point measured. At 4 months post-HSCT, naive T cell numbers were similar between ECP responders and the aGVHD corticosteroid group; however, at 8 and 12 months thymic-dependent T cell recovery was superior in the ECP responder group. These results highlight the negative impact of aGVHD and corticosteroids on thymic recovery.

### Dendritic Cell Subsets and Phenotype

**Fig. 2:**
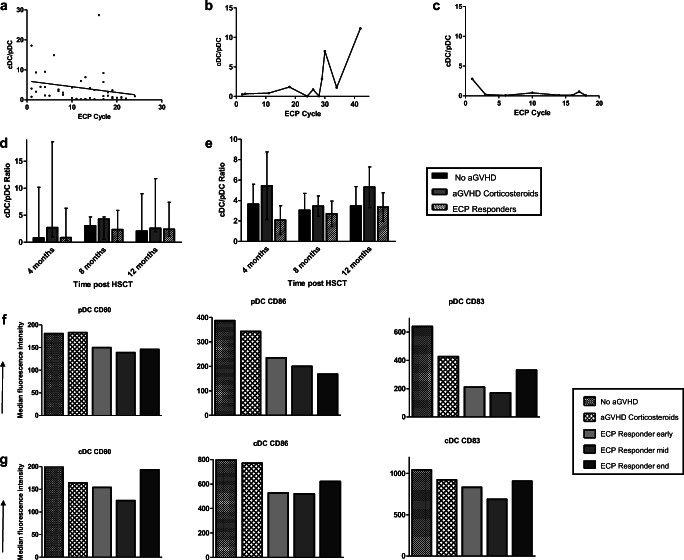
**a** A decline in the cDC/pDC ratio was observed with treatment among ECP responders (*p* value = 0.13 using linear regression analysis). Black dots shown represent cDC/pDC values in all responders during ECP treatment. **b** An incline in the cDC/pDC ratio was seen in partial responder 5. **c** cDC/pDC ratio was low throughout treatment in partial responder 6. **d** Compared to the control groups at 4, 8, and 12 months post-HSCT, the median (with range) cDC/pDC ratio was highest in the aGVHD corticosteroid group at each time point measured. Differences between the groups were not statistically significant (*p* value = 0.07). **e** A similar pattern was observed in the mean cDC/pDC ratios (error bars indicating SEM) of each group. **f**, **g** Median MFI of CD80, CD86, and CD83 expression of the ECP responders (*n* = 4) at 3 time points during treatment (early, middle, and end) was lower compared to that measured in three patients from each control group at 4 months post-HSCT

We next sought to understand mechanisms by which ECP mitigates aGVHD activity. Plasmacytoid DC numbers increased with treatment in responders (Fig. S10), with an overall decline in cDC/pDC ratio indicating a relatively higher increase in pDCs compared to cDCs (Fig. 2a), a population shown to mediate aGVHD tolerance and facilitate engraftment [20]. In contrast, the cDC/pDC ratio increased with treatment in partial responder 5 and remained persistently low in partial responder 6 (Fig. 2b, c).

To decipher the potential influence of aGVHD and corticosteroids on this cDC/pDC pattern, we compared ECP responder cDC/pDC ratios at 4, 8, and 12 months post-HSCT with the control groups (Fig. 2d–e). Median cDC/pDC ratio was highest in the aGVHD corticosteroid group at each timepoint post-HSCT. Although the range of values and lack of statistical significance indicate the need for analysis in additional patients to ascertain the validity of these findings, lower cDC/pDC values in the ECP responders, particularly at 4 (all ECP responders on treatment) and 8 months (3 ECP responders on treatment and one recently completed treatment) post-HSCT, support an ECP-induced effect, rather than a consequence of corticosteroid use or aGVHD.

Conventional DC and pDC expression of co-stimulatory markers CD80, CD86, and CD83 was measured to examine the stage of DC maturation, an important determinant of DC immune regulatory or stimulatory capacity. We observed reduced expression of co-stimulatory markers in the ECP responders (Fig. 2f–g) in comparison to expression in 3 patients from each control group at 4 months post-HSCT, indicating a more immature or tolerogenic DC phenotype at the time points measured. In contrast, co-stimulatory marker expression was higher with occasional high MFI peaks in partial responder 5 (Fig. S10).

### Regulatory T Cells

**Fig. 3:**
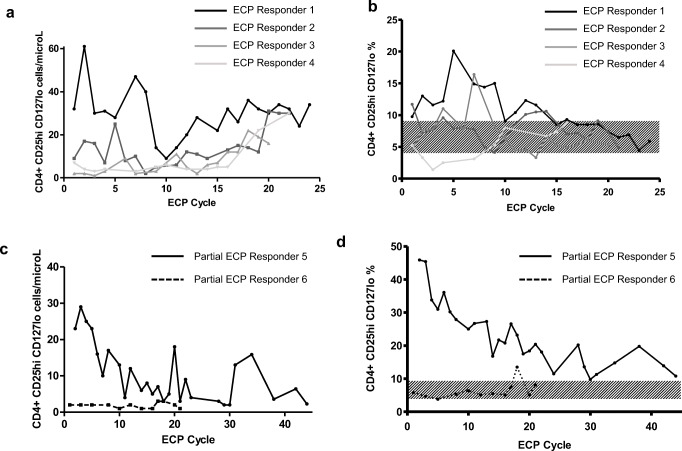

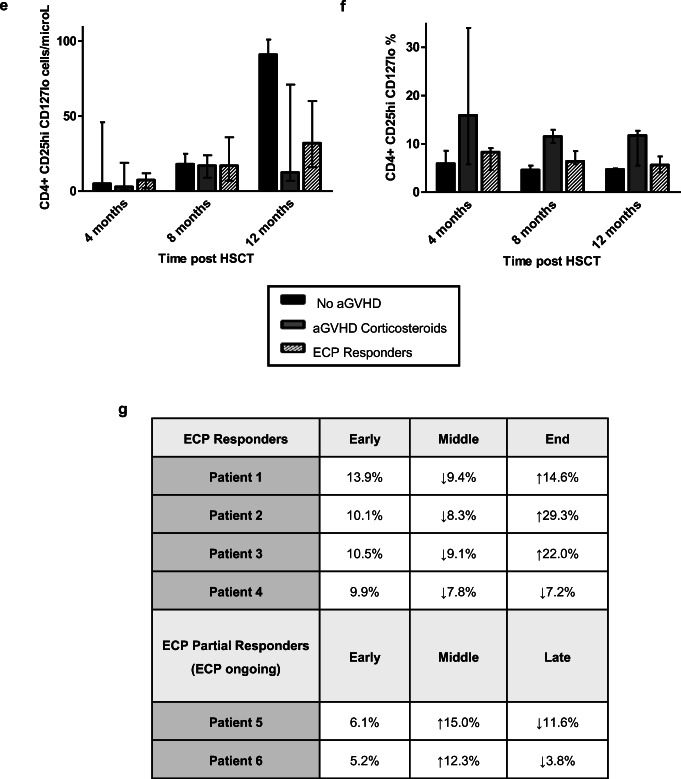
**a** Responding ECP patients demonstrated increased Treg numbers in the latter half of treatment. **b** Treg frequency intermittently increased in the responding ECP patients, but with no upward or downward trend (normal range shaded in gray). **c** Partial responder 5 demonstrated a decrease in Tregs during treatment. Partial responder 6 had low Treg numbers throughout. **d** A decline in Treg frequency was observed in partial responder 5 and remained largely within the normal range for patient 6. **e** Median numbers of Tregs (with range) were highest in the no aGVHD group followed by the ECP responders at 12 months post-HSCT. Differences between the groups were not statistically significant (*p* value = 0.49). **f** Median (with range) Treg frequency was highest in the aGVHD corticosteroid group at each time point measured. Mean values with error bars demonstrated a similar pattern (Fig. S11). **g** In the ECP responders, Teff proliferation decreased mid-ECP treatment indicating increased Treg suppression compared to early in the treatment course. The arrows indicate the change (increase or decrease) from the measurement at the previous time point

Tregs increased steadily in the ECP responders from approximately cycle 10 onwards (Fig. 3a). Responder Treg frequency increased during treatment; however, this fluctuated, without a consistent trend (Fig. 3b). Partial responder 5 demonstrated a decline in Treg number and frequency and partial responder 6 demonstrated low Treg numbers throughout and frequency mostly within the normal range (Fig. 3c, d).

In comparison to the control groups, Tregs were similar at 4 and 8 months post-HSCT, but higher in the no aGVHD group, followed by ECP responding group at 12 months (Fig. 3e, Fig. S11). Interestingly, the frequency of Tregs was highest in the aGVHD group at each time point (Fig. 3f).

Effector T cell (Teff) proliferation decreased mid-ECP treatment in the responders, indicating increased Treg suppressive capacity (Fig. 3g). Teff proliferation increased at the end of treatment in 3/4 responders indicating a reduction in Treg function at this time point but was not associated with aGVHD recurrence. In the partial responders, Teff proliferation initially increased but decreased later during treatment.

### T Cell Transcriptome Analysis

**Fig. 4:**
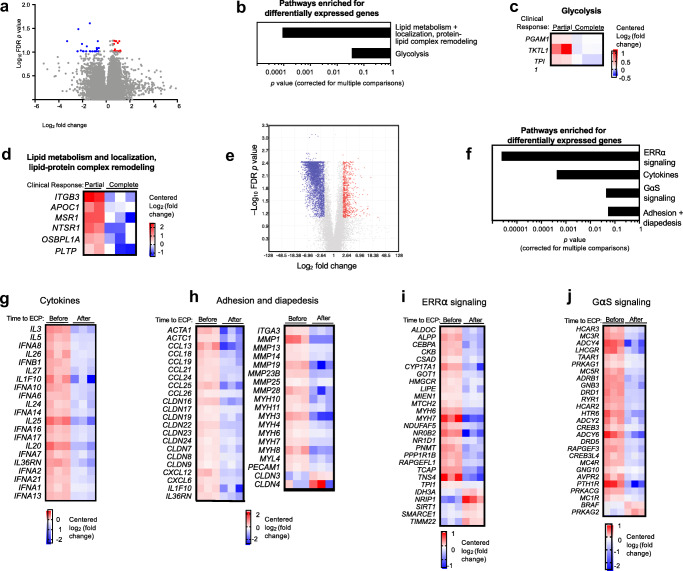
**a** Comparison of the CD3+ T cell transcriptional signature between ECP responders and partial responders after treatment identified 26 significantly downregulated genes (blue) and 24 upregulated genes (red) with a fold change of at least 1.5 times (FDR *p* value <0.1). **b**–**d** Significant enrichment of genes involved in Teff metabolism was identified, including genes important in lipid metabolism and localization, lipid-protein complex remodeling, and glycolysis. **e** T cell whole transcriptome analysis pre- and post-treatment in ECP responders identified 3333 significantly downregulated genes (blue) and 364 upregulated genes (red) with at least a two-fold difference in expression. **f**–**j** Alterations in gene expression were seen in pathways associated with cytokines, cellular adhesion, and diapedesis, and T cell activation including estrogen-related receptor alpha (ERRα) signaling and GαS signaling

Having identified differences in thymopoiesis and DC and Treg patterns, we explored differences in the CD3^+^ T cell transcriptional signature following treatment. Compared to the partial responders, ECP responders had 26 downregulated and 24 upregulated genes (>1.5-fold difference in expression, FDR < 0.05) with significant enrichment of genes in pathways important in Teff metabolism (Fig. 4a, b). Specifically, expression of genes important for lipid metabolism and localization, lipid-protein complex remodeling, and glycolysis were downregulated in responder T cells compared to partial responders after treatment (Fig. 4c, d). To identify pathways most affected by ECP treatment, we performed whole transcriptome analysis on CD3^+^ T cells from ECP responders before and after ECP treatment. CD3^+^ T cells after treatment had 3333 downregulated and 364 upregulated genes with at least a 2-fold difference in expression compared to before treatment (FDR <0.05, Fig. 4e), enriched in pathways related to estrogen-related receptor alpha (ERRα) signaling, GαS signaling, cytokines, and cellular adhesion and diapedesis (Fig. 3f–j). ERRα and GαS signaling pathways are involved in Teff activation and function. Downregulated cytokine genes included those associated with aGVHD pathogenesis [21, 22]. Downregulation of genes involved in cellular adhesion and diapedesis corresponds to the clinical resolution of inflammation observed in the ECP responders at the end of treatment.

## Discussion

We show that ECP responding patients exhibit qualitative and quantitative thymic-dependent T cell recovery, likely due to reduced aGVHD activity and immunosuppression, reinforcing previous observations [3, 16], and supporting the use of ECP as a “thymic-sparing” therapeutic strategy in aGVHD to facilitate T cell reconstitution. In addition, the slower rate of thymic-dependent T cell recovery in the aGVHD corticosteroid group highlights the negative influence of prolonged corticosteroids and the need to develop alternative “thymic-sparing” approaches.

Importantly, partial ECP responders did not demonstrate thymic recovery, at least in the time frame post-HSCT analyzed in this study. Increased IL-7 production in response to lymphopenia suggests the presence of functioning thymic epithelial cells; failure to mount IL-7 production as observed in patients 5 and 6 suggests thymic dysfunction which may reflect ongoing active thymic aGVHD and/or irreparable thymic damage. Although unclear if this is a permanent immune outcome, it raises the important question of whether multiple/prolonged insults to the thymus can cause irreversible damage. Contributing factors may include longer corticosteroid exposure, slower weaning of immunosuppression, and later ECP initiation. Patients 5 and 6 commenced ECP 173 and 142 days after HSCT respectively, in contrast to a median of 48 days in the responders, supporting previous reports that earlier ECP may produce better outcomes [23] and suggesting that limiting insult exposure may permit faster recovery of thymic function. Investigation in additional patients is needed, and in adult patients who exhibit continued but reduced thymopoiesis associated with aging [24].

Previous studies indicate a key role played by DCs and Tregs in the immunomodulating mechanisms of ECP. We demonstrate changes in DC subsets and phenotype in ECP responders supporting an immunotolerant environment and identified differences with partial responders. Comparison with control groups provide insight into the potential mechanisms of ECP independent of the impact of corticosteroids and aGVHD, but analysis in additional patients from all groups is required to confirm the findings from this small cohort. A decline in the cDC/pDC ratio in aGVHD treatment with ECP was described by Shiue et al [25] although how this shift to encourage pDC development occurs is not yet known. Reduced co-stimulatory molecule expression indicates an immature tolerogenic DC phenotype shown to be protective against aGVHD and may contribute to reduced aGVHD activity due to inadequate co-stimulatory help [26]. While these results support a potential effect of ECP on DCs, gaps remain in our knowledge regarding their function and further examination should include functional testing such as cytokine production and T cell proliferative response.

Our data demonstrate increasing Treg numbers and increased Treg suppressive capacity mid-ECP treatment in responders. Increasing Tregs coinciding with renewed naive T cell output and without a corresponding increase in frequency suggest this incline is attributable to immune reconstitution and egress of thymic-derived Tregs. However, activated Tregs may be primarily located in sites of tissue inflammation, particularly in the early stage of ECP treatment. Examination of Tregs in aGVHD-affected tissue and specifically measuring CD45RA^+^CD31^+^ Tregs would be valuable to elucidate Treg patterns and origin [27]. Certain Treg subpopulations are associated with greater suppressive capacity, which could account for the increased Treg suppressive function observed; a more detailed Treg analysis is needed to identify if expansion of certain subsets occurs in ECP such as IL-10-producing Tr1 Tregs. Higher Treg frequencies in the aGVHD corticosteroid group and partial responder 5 may be related to longer corticosteroid exposure [28]. However, partial responder 6, who was also exposed to prolonged corticosteroids, did not demonstrate elevated Treg frequencies suggesting involvement of other factors. Further examination of the impact of corticosteroids on Tregs would help to understand the independent effect of ECP.

ECP responders exhibited significant downregulation in gene pathways important in Teff metabolism compared to partial responders, pathways essential for allogeneic Teffs driving aGVHD [29–31]. Although partial responders demonstrated some clinical progress, these data indicate that their T cells retain transcriptional characteristics known to be important for aGVHD. Further evaluation in ECP responders before and after treatment displayed significant changes in ERRα and GαS signaling pathways which have not been highlighted in prior studies of T cells from patients or animal models with aGVHD. ERRα, a transcriptional regulator of cellular metabolism, increases Teff activation and proliferation by increasing expression of genes important for glycolysis and mitochondrial function [32, 33]. ERRα deficiency reduces Teff proliferation and function in experimental autoimmune encephalitis [33] and ERRα downregulation in the setting of ECP therapy may reduce activated Teff generation. GαS signaling is important for Th1 and Th17 differentiation and function but dispensable in Treg generation [34]. Deletion of GαS in CD4^+^ T cells results in reduced generation of cAMP, decreased calcium flux, and the inability to induce colitis in an adoptive transfer model [34], underscoring the importance of this pathway in CD4^+^ T cell–driven inflammation. Reduced T cell activation via these pathways may indirectly promote naive T cells and Tregs by reducing inflammation and facilitating immune reconstitution, and directly promote T cell differentiation towards a Treg phenotype. Further investigation is needed in additional patients to validate these findings and their functional consequences and to determine if this transcriptional signature is unique to ECP therapy.

The known importance of T cell chemotaxis in aGVHD pathogenesis [35–38] supports our observation of reduced gene expression in pathways involved in T cell migration, adhesion, and diapedesis in parallel with aGVHD resolution post-ECP. Reduced cytokine gene expression included type 1 interferons which may contribute to reduced alloreactive CD8^+^ responses. Type 1 interferon signaling is known to increase alloreactive cytotoxic T cell expansion, cross-present host antigens to CD8^+^ T cells, and activate bystander cells [39]. Further investigation at protein and functional levels, including correlation with T cell cytokine secretion and quantitative cellular adhesion assay would be valuable to validate these transcriptomic findings.

Study limitations include the small size and heterogeneity of the cohort. Examination of additional patients and further investigations, as outlined, are needed. Investigation of larger patient and control cohorts is essential to adjust for differences in characteristics between these clinically heterogenous groups and permit clearer interpretation of results. Limited blood volumes from control groups precluded the ability to perform all analyses on these patients, which is needed for comprehensive comparison with the ECP patients.

In conclusion, these data indicate that successful ECP associates with thymopoietic recovery in children with aGVHD and can be used as a strategy to promote immune reconstitution, especially when used early. Correlation with reconstitution of other immune cell groups and long-term follow-up is important. We demonstrate, for the first time, differences at a cellular and transcriptional level in patients with a partial ECP response. Alterations in DCs and Tregs suggest that peripheral tolerance is augmented in ECP responders and, as these patterns were not observed in partial responders, lack of upregulation could contribute to an incomplete response. We observed distinct T cell transcriptome changes in responders, with decreased expression of genes important for T cell activation, even in the absence of immunosuppression. As ECP is expensive, time-consuming, and requires central venous access in children, identifying those who will benefit from treatment is an important goal. Due to the nature of this therapy and population, investigation in large numbers is challenging. These data will direct further investigation in additional patients to validate findings and allow statistical inference. In addition, extension of investigations to include detailed Treg phenotyping, DC functional testing, and analysis of functional downstream consequences of T cell transcriptome changes is needed, as well as further longitudinal analysis to explore the longevity of cellular and transcriptomic alterations observed. Confirmation of specific biomarkers will assist clinical decision-making to determine who can benefit from ECP treatment.

## Supplementary Information

ESM 1 (PDF 3266 kb)

## Data Availability

Available upon request from the corresponding author.
