# Supplementary material for: Thymopoiesis, Alterations in Dendritic Cells and Tregs, and Reduced T Cell Activation in Successful Extracorporeal Photopheresis Treatment of GVHD
Source: J Clin Immunol. 2021 Mar 2;41(5):1016–30. doi: 10.1007/s10875-021-00991-y (PMC8249294; doi:10.1007/s10875-021-00991-y)
Supplement: Supplementary file 1 — (PDF 3266 kb) [file 10875_2021_991_MOESM1_ESM.pdf]

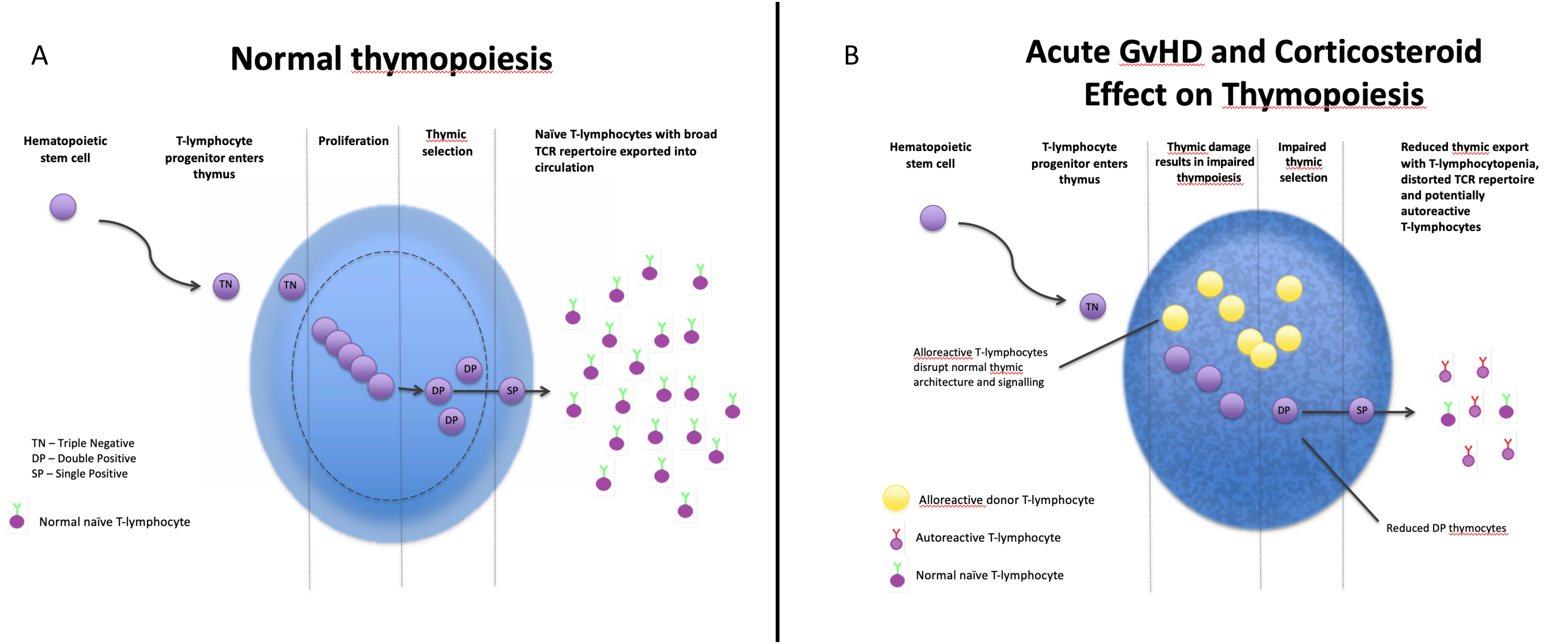

Figure S1

(A) Normal thymopoiesis results in export of naïve T-lymphocytes with a broad T-cell receptor (TCR) repertoire into the circulation. (B) Thymic damage occurs secondary to aGvHD and from corticosteroids used in the treatment of aGvHD, causing impaired thymopoiesis, with reduced thymic export and a distorted TCR repertoire with potentially autoreactive thymocytes escaping negative selection. Figure taken from Flinn AM, Gennery AR. Extracoporeal photopheresis treatment of acute graft-versus-host disease following allogeneic haematopoietic stem cell transplantation. F1000Res. 2016 Jun 27;5: F1000 Faculty Rev-1510.

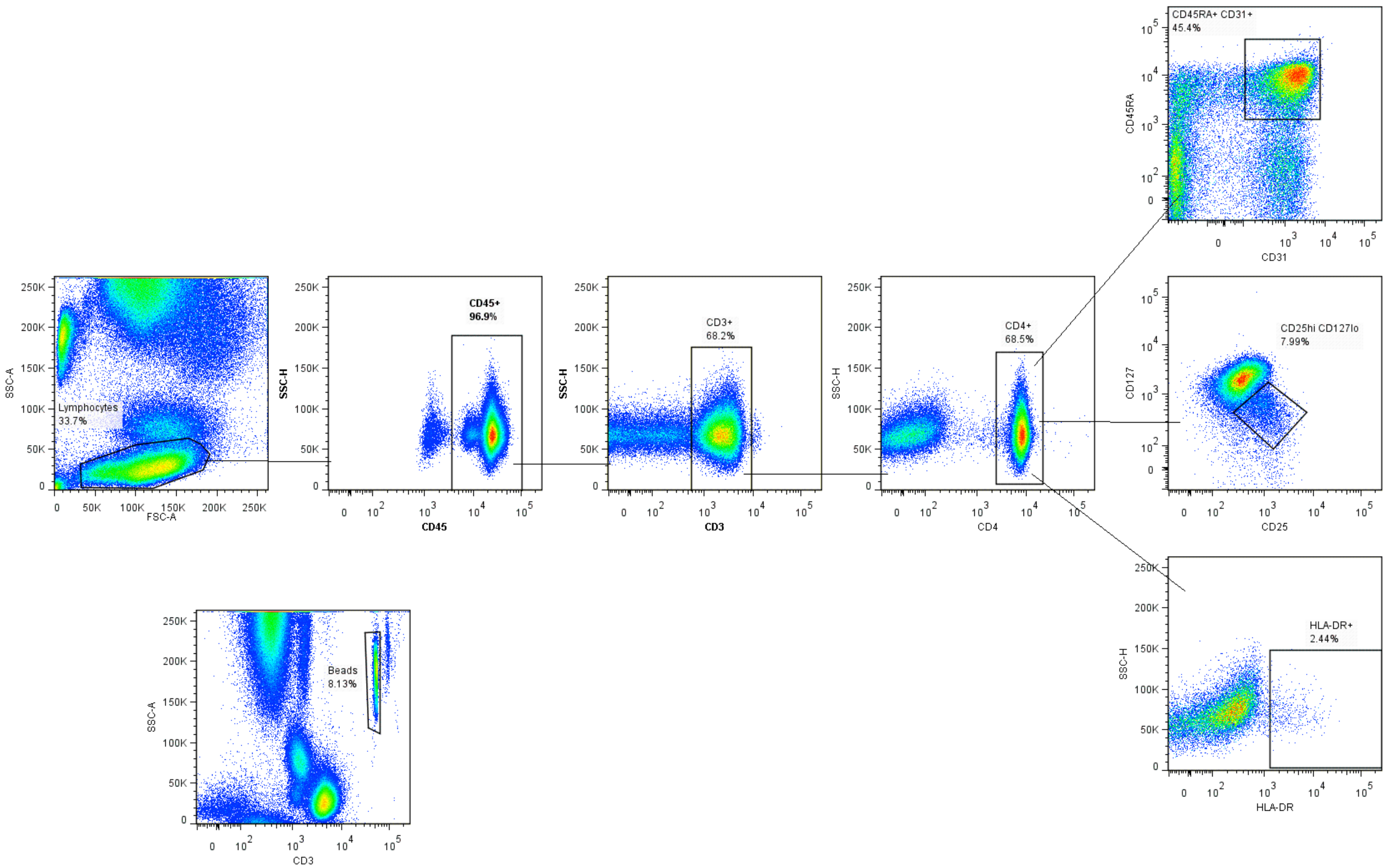

Figure S2

Gating strategy to isolate naive T-cells ($CD3^+CD4^+CD45RA^+CD31^+$), Tregs ($CD3^+CD4^+CD25^{hi}CD127^{lo}$) and activated T-cells ($CD3^+CD4^+HLA\text{-}DR^+$).

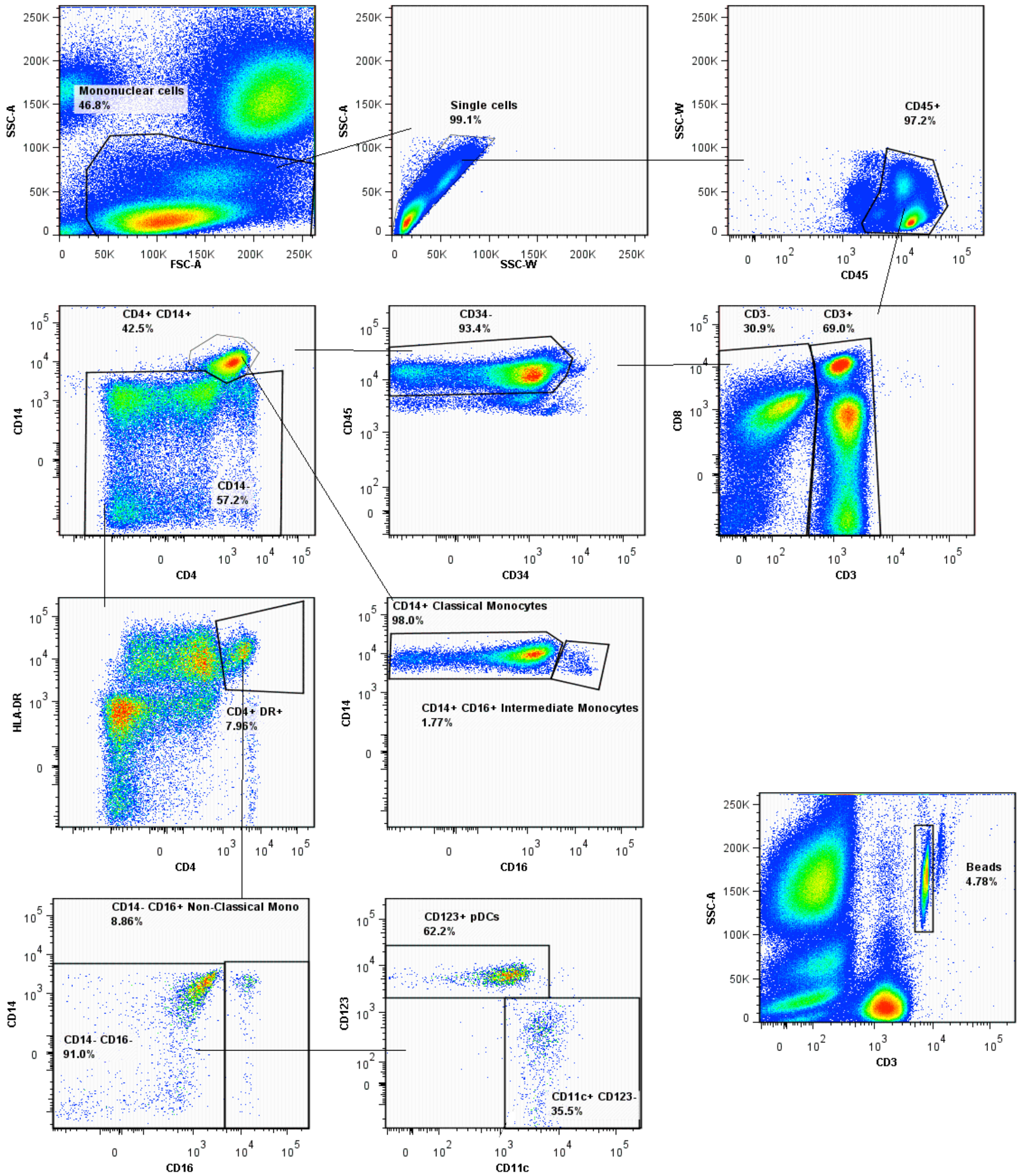

Figure S3

Gating strategy for DC enumeration (cDCs; CD45+CD3-CD34-CD14-DR+CD4+CD16-CD11c+ and pDCs; CD11c-CD123+)

A

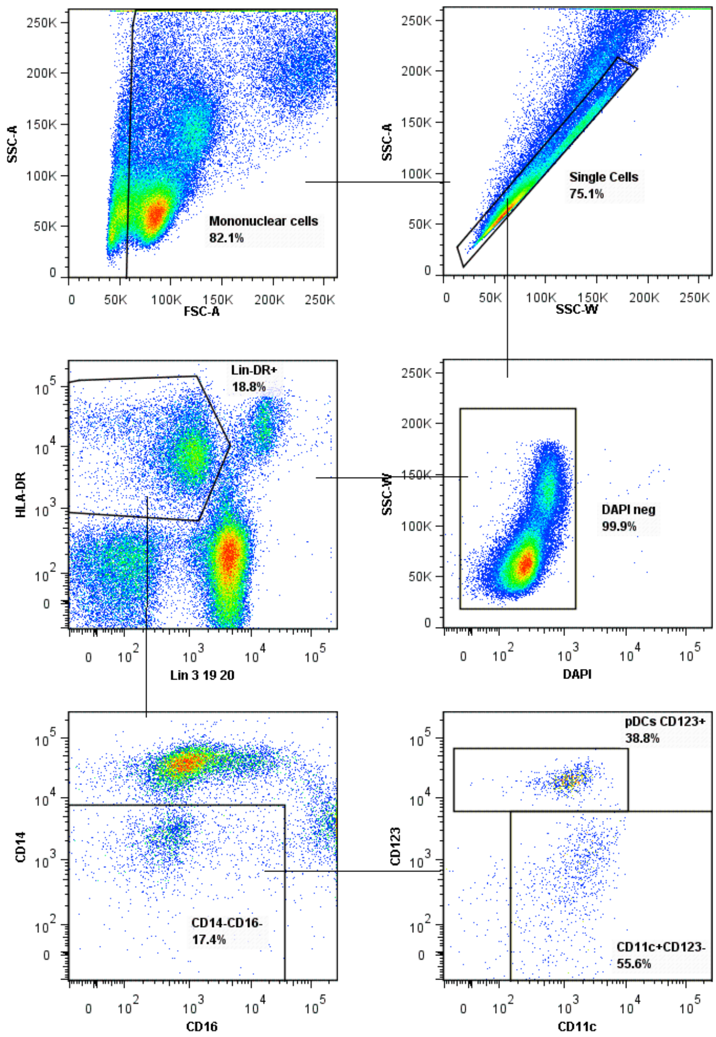

B

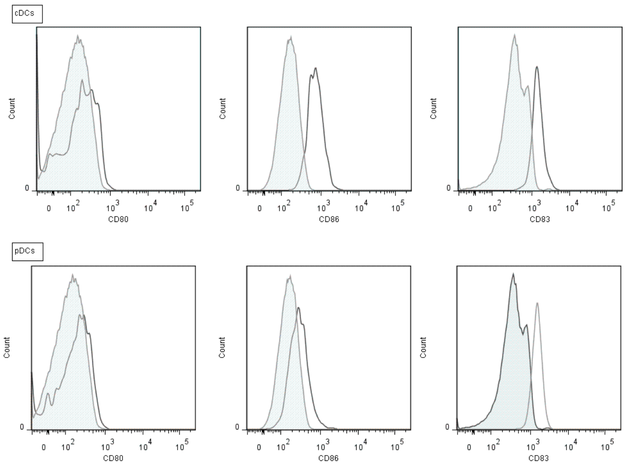

Figure S4

(A) Gating strategy to isolate cDCs and pDCs and (B) expression of CD86, CD80 and CD83 (isotype controls shown in shaded area).

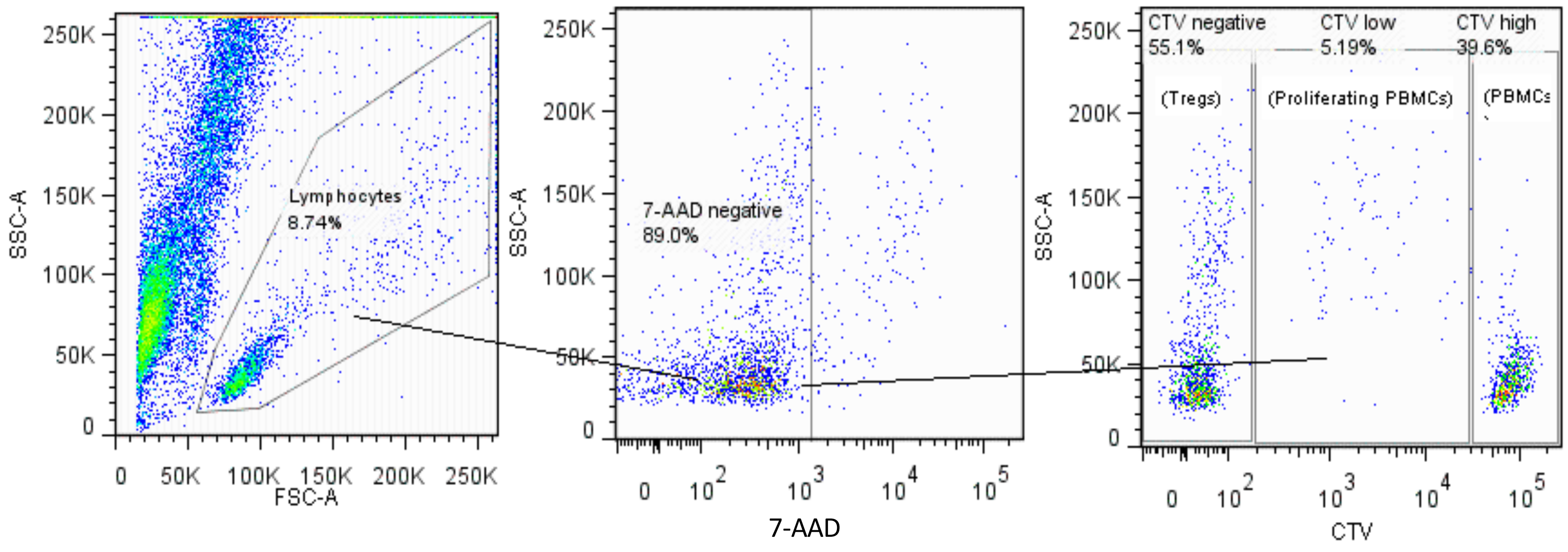

Figure S5

The CTV-negative gate represents unstained Tregs and the CTV-high gate represents CTV-stained PBMCs. The CTV-low gate represents the proliferating T-cells. Treg suppressive capacity was measured in the ECP patients by comparing the percentage of T-cell proliferation measured in the CTV-low gate at three timepoints during treatment (beginning, middle and end).

Cβ	GGGTGTGGGAGATCTCTGC
Cβ (FAM labelled)	ACACAGCAGCCTCGGGTGGG
Vβ1	CCGCACAACAGTTCCCTGACTTGC
Vβ2	CACAACTATGTTTTGGTATCGTC
Vβ3	CGCTTCTCCCTGATTCTGGAGTCC
Vβ4	TTCCCATCAGCCGCCCAAACCTAA
Vβ5	GATCAAAACGAGAGGACAGC
Vβ6a	GATCCAATTTCAGGTCATACTG
Vβ6b1	CAGGGSCCAGAGTTTCTGAC
Vβb2	CAGGGCTCAGAGGTTCTGAC
Vβ7	CCTGAATGCCCCAACAGCTCT
Vβ8	GGTACAGACAGACCATGATGC
Vβ9	TTCCCTGGAGCTTGGTGACTCTGC
Vβ11	GTCAACAGTCTCCAGAATAAGG
Vβ12	TCCYCCTCACTCTGGAGTC
Vβ13a	GGTATCGACAAGACCCAGGCA
Vβ13b	AGGCTCATCCATTATTCAAATAC
Vβ14	GGGCTGGGCTTAAGGCAGATCTAC
Vβ15	CAGGCACAGGCTAAATTCTCCCTG
Vβ16	GCCTGCAGAACTGGAGGATTCTGG
Vβ17	TCCTCTCACTGTGACATCGGCCCA
Vβ18	CTGCTGAATTTCCCAAAGAGGGCC
Vβ20	TGCCCAGAATCTCTCAGCCTCCA
Vβ21	GGAGTAGACTCCACTCTCAAG
Vβ22	GATCCGGTCCACAAAGCTGG
Vβ23	ATTCTGAACTGAACATGAGCTCCT

Figure S6

Primers used for TCR complementarity-determining region 3 spectratyping.

a

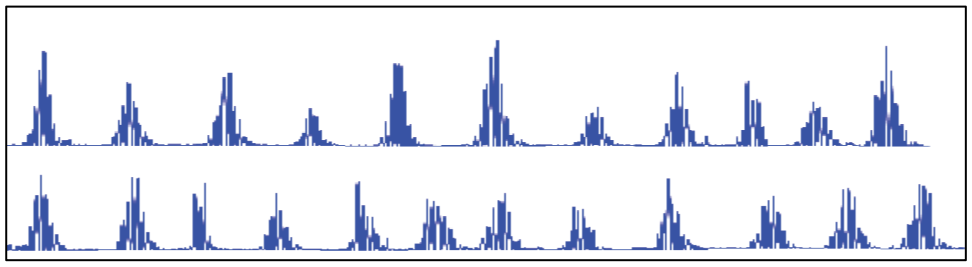

b

**Normal**	≥22 families having between 5-12 peaks with less than 3 non-Gaussian families and ≤3 large oligoclonal or monoclonal peaks.
**Abnormal**	All families present with ≥20 having 5-12 peaks, ≥3 but less than 8 families have a non-Gaussian distribution, >3 but ≤6 large oligoclonal or monoclonal peaks.
**Highly abnormal**	Some absent families or <20 families having 5-12 peaks and ≥8 families having a non-Gaussian distribution, >6 large oligoclonal or monoclonal peaks.

Figure S7

(a) Healthy control electropherograms (n=3) were first analyzed to develop a scoring system as shown in (b) based on (1) the typical number of peaks per Vβ family, (2) the number of Gaussian families per spectratype and (3) the presence of any large oligoclonal or monoclonal peaks per spectratype. Patient electropherograms were then scored as normal, abnormal or highly abnormal. This provided a subjective description of the TCR spectratype over time.

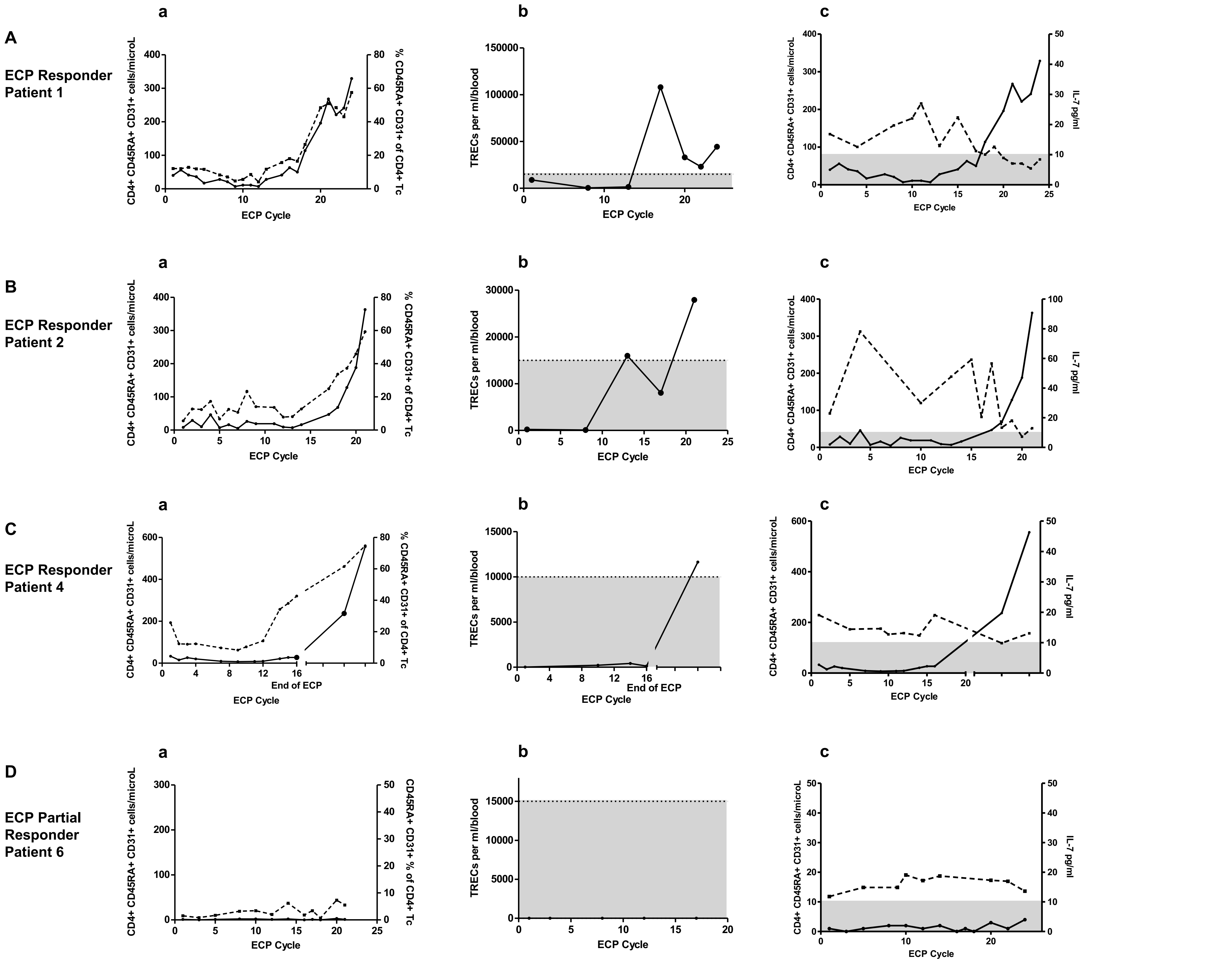

Figure S8

In the ECP responders (A-C) (a) increased frequency (dotted line) and absolute number (continuous line) of naive T-cells and (b) TRECs (shaded area represents the upper limit of the normal range for age) was observed. (c) An inverse relationship between IL-7 (dotted line) and number of naive T-cells (continuous line) was seen. (D) Partial responder 6 showed (a) ongoing negligible number (continuous line) and frequency (dotted line) of naive T-cells and (b) TRECs. (D-c) An inverse relationship with serum IL-7 was not observed. Data from ECP responder 3 and partial responder 5 are shown in Figure 1.

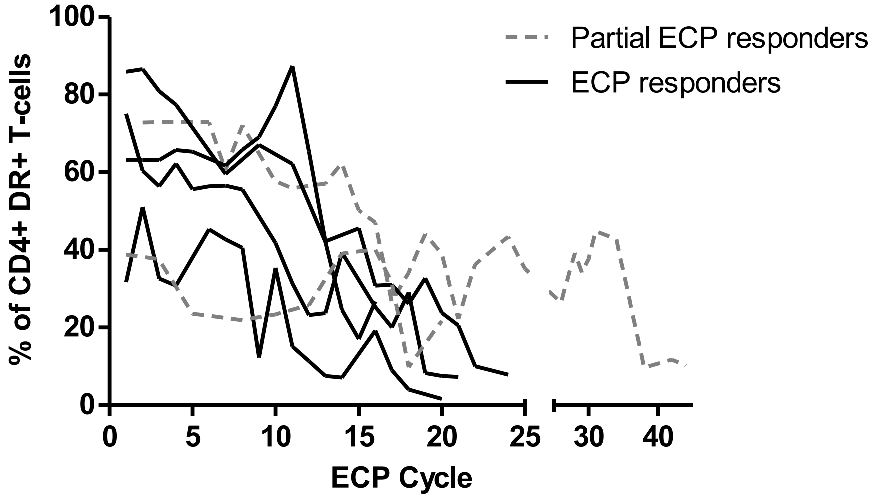

Figure S9

ECP responders (continuous black lines) and partial ECP responders (dotted grey lines) demonstrated a decline in the frequency of activated DR+ T-cells with progression of ECP treatment.

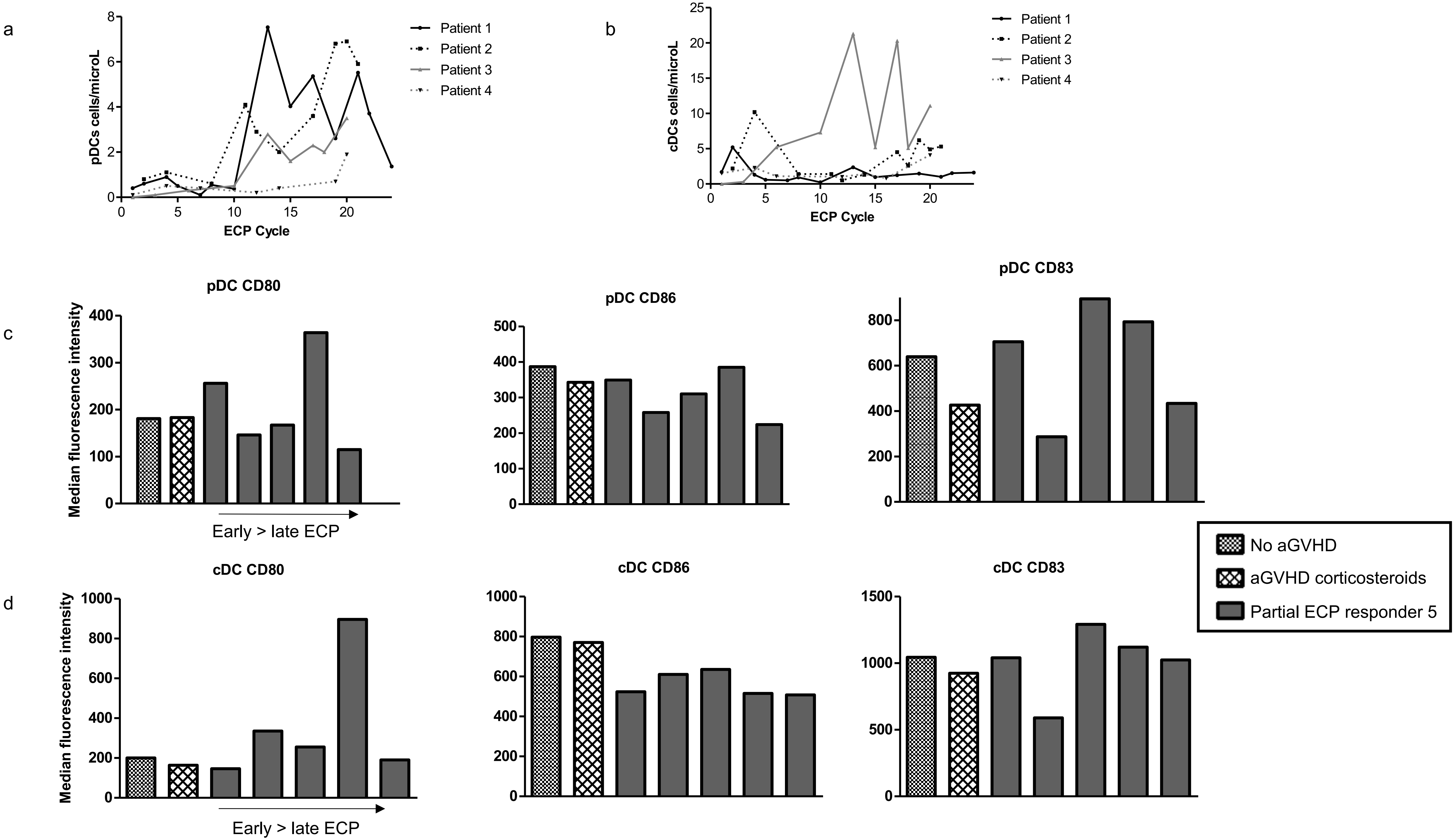

Figure S10

(a) ECP responders (patients 1-4) demonstrated an overall incline in pDCs. (b) Conventional DCs demonstrated a more variable pattern but increased in 3 patients late in the treatment course and remained static in patient 1. Plasmacytoid DC (c) and cDC (d) expression of co-stimulatory markers CD80, CD86 and CD83 was measured in partial responder 5 at increasing timepoints during ECP treatment and demonstrated a different pattern to that observed in the ECP responders (Figure 2f–g), with higher MFI values and occasional high MFI peaks compared to the control groups.

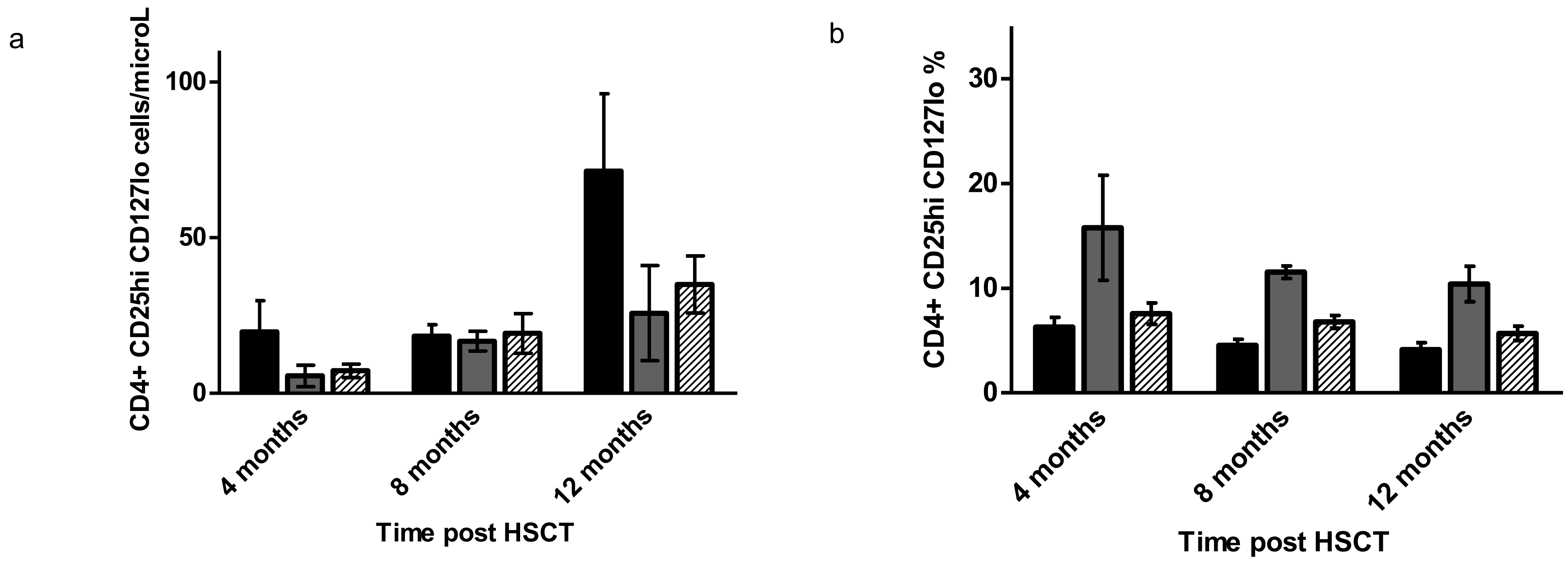

Figure S11

(a) Mean absolute number of Tregs (error bars indicating SEM) was highest in the no aGVHD group followed by the ECP responder group at 12 months post-HSCT. (b) Mean (with error bars) Treg frequency was highest in the aGVHD corticosteroid group at each timepoint measured.

Patient	Pre ECP result	Description	Post ECP result	Description
Patient 1	Highly abnormal	22 families with ≥5 peaks, 15 oligo/monoclonal peaks, 9 non-Gaussian families	Abnormal but improved	22 families with ≥5 peaks, 2 non-Gaussian families, 6 oligo/monoclonal peaks peaks.
Patient 2	Highly abnormal	23 families with ≥5 peaks, 11 oligo/monoclonal peaks, 3 non-Gaussian families	Normal	All families present with between 5-12 peaks, 2 families with non-Gaussian distributions, 3 monoclonal peaks
Patient 3	Highly abnormal	22 families with ≥ 5 peaks, 9 oligo/monoclonal peaks 4 non-Gaussian families	Normal	22 families with ≥5 peaks, all Gaussian distributions, 2 monoclonal peaks
Patient 4	Highly abnormal	23 families with ≥5 peaks, 13 oligo/monoclonal peaks, 12 non-Gaussian families	Abnormal but improved	22 families with ≥5 peaks, 2 families with non-Gaussian distributions, 11 monoclonal peaks
Patient 5	Highly abnormal	22 families with ≥5 peaks, 17 oligo/monoclonal peaks, 10 non-Gaussian families	ECP ongoing<br>Cycle 44: highly abnormal	One family < 5 peaks. All other families present with between 5-12 peaks, 2 families with non-Gaussian distributions, frequent (x15) monoclonal peaks.
Patient 6	Highly abnormal	23 families ≥ 5 peaks, 16 oligo/monoclonal peaks, 8 non-Gaussian families	ECP ongoing	ECP ongoing

Table S1

TCR repertoires were scored as normal, abnormal or highly abnormal (scoring system shown in Online Resource 3), providing a visual description. All patients had a highly abnormal TCR repertoire at the beginning of ECP treatment, consistent with poor thymic output and a restricted TCR repertoire. ECP responders demonstrated improvement in the TCR repertoire indicating qualitative improvement in the T-cell compartment, consistent with improved thymic output. No improvement in the TCR repertoire was seen in partial responder 5 despite prolonged ECP. Partial responder 6 demonstrated a highly abnormal TCR spectratype before ECP and remains on ongoing treatment.
